# Effect of Glucagon-like Peptide-1 Receptor Agonism on Aortic Valve Stenosis Risk: A Mendelian Randomization Analysis

**DOI:** 10.3390/jcm13216411

**Published:** 2024-10-26

**Authors:** Paschalis Karakasis, Dimitrios Patoulias, George Giannakoulas, Marios Sagris, Panagiotis Theofilis, Nikolaos Fragakis, Giuseppe Biondi-Zoccai

**Affiliations:** 1 Second Department of Cardiology, Hippokration General Hospital, Aristotle University of Thessaloniki, 54642 Thessaloniki, Greece; fragakis.nikos@googlemail.com; 2 Department of Hygiene, Social-Preventive Medicine & Medical Statistics, Medical School, Aristotle University of Thessaloniki, University Campus, 54642 Thessaloniki, Greece; 3 Second Propedeutic Department of Internal Medicine, Hippokration General Hospital, Aristotle University of Thessaloniki, 54642 Thessaloniki, Greece; dipatoulias@gmail.com; 4 First Department of Cardiology, AHEPA University Hospital, Aristotle University of Thessaloniki, 54636 Thessaloniki, Greece; g.giannakoulas@gmail.com; 5 School of Medicine, Hippokration General Hospital, National and Kapodistrian University of Athens, 15772 Athens, Greece; masagris1919@gmail.com (M.S.); panos.theofilis@hotmail.com (P.T.); 6 Department of Medical-Surgical Sciences and Biotechnologies, Sapienza University of Rome, 04100 Latina, Italy; gbiondizoccai@gmail.com; 7 Maria Cecilia Hospital, GVM Care & Research, 48033 Cotignola, Italy

**Keywords:** aortic valve stenosis, diabetes, obesity, glucagon-like peptide-1

## Abstract

**Background:** Aortic valve repair is currently the only effective treatment for calcific aortic valve stenosis (CAVS), as no pharmacological therapies exist to prevent or slow its progression. Recent promising results showed that glucagon-like peptide-1 (GLP-1) attenuates the calcification of aortic valve interstitial cells. Therefore, we conducted a two-sample Mendelian randomization analysis to investigate the effect of GLP-1 receptor agonism (GLP-1Ra) on the risk of CAVS. **Methods:** The inverse variance weighted (IVW) method was used to obtain the primary causal inference, and several sensitivity analyses, including MR-Egger, were performed to assess the robustness of the results. **Results:** Based on the IVW estimates, the GLP-1Ra showed a neutral effect on the risk of CAVS (odds ratio [OR] per 1 mmol/mol decrease in glycated hemoglobin = 0.87, 95% CI = [0.69, 1.11], *p* = 0.259; I^2^ = 4.5%, Cohran’s Q = 2.09, heterogeneity *p* = 0.35; F statistic = 16.8). A non-significant effect was also derived by the sensitivity analyses. No evidence of horizontal pleiotropy was identified. **Conclusions:** GLP-1Ra was not significantly associated with the risk of CAVS. Furthermore, pragmatically designed studies are required to evaluate the effect of GLP-1Ra on the clinical course of CAVS in different patient subgroups.

## 1. Introduction

Calcific aortic valve stenosis (CAVS) represents the predominant type of incident valvular heart disease in high-income populations [1]. Among individuals aged over 75 years old, CAVS prevalence ranges from 10% to 15%, with an anticipated more than twofold increase by 2040 [2]. Despite the effectiveness of aortic valve replacement for severe CAVS cases, there are currently no treatments available to prevent progression to valve replacement [3]. Moreover, uncertainty persists regarding which patients are at high risk of a severe prognosis. A genetic component contributes to the etiology of CAVS, as siblings of CAVS patients face a more than fourfold increased risk [4]. Previous genome- and transcriptome-wide association studies have identified genetic loci associated with CAVS, including apolipoprotein(a) [5] and interleukin-6 [6]. Additionally, Mendelian randomization (MR) investigations have supported a causal contribution of low-density lipoprotein cholesterol (LDL-C) [7], lipoprotein(a) [8], and body mass index (BMI) [9] to CAVS, suggesting that susceptibility to CAVS is partially mediated by lipid metabolism and inflammation.

Glucagon-like peptide-1 (GLP-1) is a peptide hormone derived from the enzymatic breakdown of proglucagon, synthesized primarily by L-cells in the intestinal mucosa, α-cells in the pancreatic islets, and neurons in the nucleus of the solitary tract [10]. These enteroendocrine L-cells, located in the distal jejunum, ileum, and colon, secrete GLP-1 in response to nutrient intake and neuroendocrine stimuli. GLP-1 is produced from the preproglucagon precursor, which undergoes proteolytic processing within L-cells, yielding two major bioactive forms, which are GLP-1(1–37) and the more predominant GLP-1(7–36) amide or GLP-1(7–37) peptide variants [11]. As an incretin hormone, GLP-1 plays a crucial role in maintaining glucose homeostasis by modulating insulin and glucagon secretion in response to elevated blood glucose levels [12,13]. Despite its critical physiological functions, GLP-1 has an inherently short half-life, persisting in circulation for only 1–2 min under normal physiological conditions [14]. This rapid inactivation is primarily due to enzymatic degradation by dipeptidyl peptidase IV (DPP-4), which diminishes its biological activity [14].

The action of GLP-1 is mediated through the GLP-1 receptor (GLP-1R), a member of the G-protein-coupled receptor (GPCR) family, which exhibits a high affinity for GLP-1 [15]. GLP-1R is widely expressed beyond the pancreas, including in various organs and tissues throughout the body, reflecting its broad physiological roles that extend beyond glucose regulation [16,17]. The knockout (KO) of GLP-1R in animal models highlights its importance, as it leads to significant metabolic and physiological changes, including increased appetite, weight gain, impaired pancreatic function, and disruptions in insulin and glucagon balance [18,19]. Moreover, GLP-1R is expressed in the cardiovascular system and central nervous system (CNS), where its absence can result in cardiovascular dysfunction and altered cognitive, behavioral, or mood states [20,21].

Glucagon-like peptide-1 receptor agonists (GLP-1Ras) are pharmacologically engineered analogs of GLP-1, developed through precise structural modifications that enhance their resistance to DPP-4-mediated degradation [22]. These synthetic proteins, which partially or fully mimic the amino acid sequence of endogenous GLP-1, exhibit extended half-lives and enhanced biological activity compared to native GLP-1 [22]. By replicating the glucose-lowering effects of natural GLP-1, GLP-1Ras help to regulate blood glucose levels without significantly increasing the risk of hypoglycemia. Lately, GLP-1Ras have captured scientific interest for their remarkable cardiovascular effects, with proven benefits to reduce cardiovascular risk in high-risk individuals with DM, independent of baseline glycated hemoglobin [23,24,25]. The potential therapeutic applications of GLP-1Ras are expanding, with ongoing research suggesting benefits across a broad range of diseases [26]. Recent studies have shown that the benefits of GLP-1Ras extend beyond atherosclerotic disease protection, indicating potential therapeutic applications across a broader spectrum of cardiovascular conditions, including obesity-related heart failure [27] and atrial fibrillation [28,29]. Of note, a recent study showed that decreased levels of GLP-1 are associated with CAVS, reporting that GLP-1 suppresses the calcification of aortic valve interstitial cells [30]. Similar favorable effects were also reported for liraglutide, a long-acting GLP-1 receptor agonist, which was associated with the attenuation of aortic valve calcification in preclinical models [31]. In light of these findings, we conducted an MR analysis to investigate the association between GLP-1 receptor agonism (GLP-1Ra) and the genetically predicted risk of CAVS.

## 2. Materials and Methods

**Figure 1:**
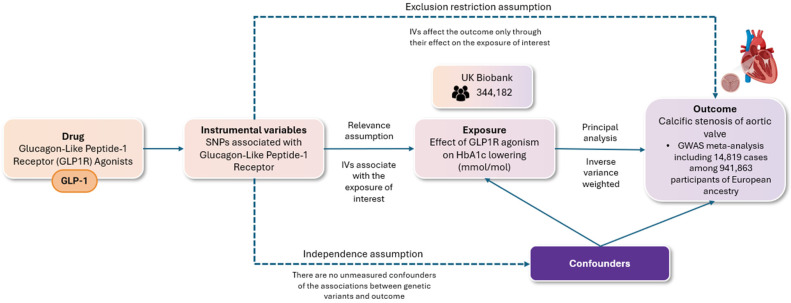
The flowchart depicts a two-sample randomization analysis assessing the impact of glucagon-like peptide-1 receptor agonism on the risk of aortic valve stenosis. The following key assumptions were rigorously evaluated: (i) the relevance assumption, ensuring that the selected genetic variants are indeed associated with the specified risk factor; (ii) the independence assumption, which posits the absence of unmeasured confounders that could affect the relationship between the genetic variants and the outcome; and (iii) the exclusion restriction assumption, which requires that the genetic variants influence the outcome exclusively through their effect on the risk factor of interest. Single nucleotide polymorphisms (SNPs) associated with GLP-1 receptor (GLP-1R) agonists were used as instrumental variables for a two-sample Mendelian randomization (MR) analysis. Genome-wide significant variants (*p* < 5 × 10^−8^) in the GLP-1 receptor gene were selected, excluding those with linkage (r^2^ < 0.1). These variants were linked to type 2 diabetes risk and glycated hemoglobin levels (HbA1c). The main analysis employed inverse variance weighting (IVW), with additional methods (weighted median, MR-Egger) to test robustness and check for pleiotropy.

Our study adhered to the Strengthening the Reporting of Observational Studies in Epidemiology using Mendelian randomization (STROBE-MR) guidelines (Supplemental Table S1). We utilized a two-sample Mendelian randomization (MR) approach to assess the validity of causal relationships, ensuring compliance with three fundamental assumptions, namely (i) the relevance assumption, which requires that the genetic variants are associated with the risk factor in question; (ii) the independence assumption, which holds that there are no unmeasured confounding factors influencing the relationship between the genetic variants and the outcome; and (iii) the exclusion restriction assumption, which stipulates that the genetic variants impact the outcome solely through their effect on the risk factor of interest. The methodology for conducting the present MR study, along with the respective assumptions, is illustrated in Figure 1.

Single nucleotide polymorphisms (SNPs), occurring in more than 1% of the population, were employed as instrumental variables to perform the two-sample MR analysis. First, we selected and validated genetic variants with an established association with GLP-1Ra [32]. In summary, we identified genetic proxies for the effects of GLP-1 receptor agonists (GLP-1Ras) as genome-wide significant variants (*p* < 5 × 10^−8^) that were uncorrelated (r^2^ < 0.1) within the GLP-1 receptor gene (genomic position on build GRCh37/hg19: chromosome 6: 39,016,574–39,055,519). These variants were associated with liability for type 2 diabetes mellitus in the largest published genome-wide association study meta-analysis, which included 228,499 cases and 1,178,783 controls, predominantly of European ancestry (79%) [33]. Furthermore, these variants exhibited directionally concordant and nominally significant associations (*p* < 0.05) with glycated hemoglobin levels in the UK Biobank (*n* = 344,182) [34]. All subsequent analyses were weighted according to the variant association with glycated hemoglobin (mmol/mol). Data for AS were obtained from a GWAS meta-analysis of 6 cohorts, including 14,819 cases among 941,863 participants of European ancestry [35]. F statistics were utilized to assess the strength of each SNP as an instrumental variable. SNPs with F values < 10 were excluded from the analyses to reduce potential bias arising from weak instruments (relevance assumption).

For each variant utilized in the MR analysis, we harmonized the genetic associations with both the exposure and the outcome by aligning effect alleles. Consequently, no exclusions were made for palindromic variants. The main analysis employed the inverse variance weighted (IVW) method, as it can offer the most accurate and robust estimates when all genetic variants are valid instruments [36], orientating estimates to a reduction in glycated hemoglobin (i.e., the direction of the drug effect). The Cochrane’s Q statistic was used for the global test of heterogeneity to evaluate heterogeneity among the genetic instruments. To evaluate the robustness of our findings concerning the weighting of variants based on their associations with glycated hemoglobin, we performed additional analyses using weights derived from the log odds of type 2 diabetes mellitus liability. Furthermore, we replicated the analysis utilizing simple and weighted median methods, as well as the MR-Egger approach, to explore the possibility of horizontal pleiotropy that could potentially bias the inverse variance weighted (IVW) estimates. The results are reported as odds ratios (ORs), along with their corresponding confidence intervals (CIs). A two-tailed *p*-value less than 0.05 was considered statistically significant. All analyses were carried out using the *TwoSampleMR, MendelianRandomization*, and *MR-PRESSO* packages in R software v. 4.2 (R Foundation for Statistical Computing, Vienna, Austria).

## 3. Results

**Figure 2:**
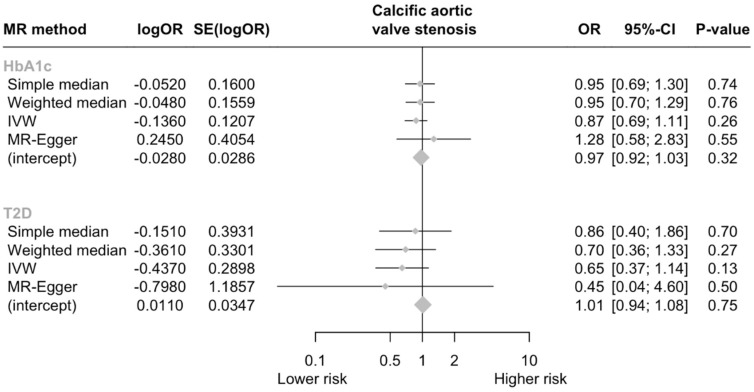
Forest plot of MR estimates. Abbreviations: HbA1c, glycated hemoglobin A1c; T2D, type 2 diabetes; CI, confidence interval; OR, odds ratio.

In total, three SNPs were identified as genetic instruments for GLP-1Ra (Table 1). The genetically proxied GLP-1Ras had a non-significant effect on the risk of CAVS (odds ratio [OR] per 1 mmol/mol decrease in glycated hemoglobin = 0.87, 95% CI = [0.69, 1.11], *p* = 0.259; I^2^ = 4.5%, Cohran’s Q = 2.09, heterogeneity *p* = 0.35; F statistic = 16.8). A non-significant effect was also derived by the MR-Egger analysis (*p* = 0.546). No evidence of horizontal pleiotropy was detected (Egger intercept = −0.028, *p* = 0.324). Analyses weighting the genetic proxies for GLP-1Ra by type 2 diabetes mellitus liability yielded similar results with the main analysis (OR per log odds increase in type 2 diabetes mellitus liability = 0.65, 95% CI = [0.37, 1.14], *p* = 0.501; Figure 2).

## 4. Discussion

Valvular endothelial cells (VECs) and valvular interstitial cells (VICs) are the primary cell types responsible for the structural integrity and functional performance of valve cusps. Increasing evidence indicates that CAVS initiates with VEC dysfunction, followed by lipid accumulation and inflammation, leading to the osteogenic differentiation and calcification of VICs as the disease advances [37,38,39]. Among these pathological processes, VEC dysfunction and VIC calcification are recognized as key features of CAVS [38]. Various signaling pathways and regulatory genes have been implicated in these mechanisms. For example, activation of the NOTCH1 signaling pathway inhibits VIC calcification by downregulating Runt-related transcription factor 2 (Runx2) and osteocalcin expression, while mutations in NOTCH1 are associated with CAVS in both human and murine models [40,41,42]. Additionally, activation of the nuclear factor kappa-light-chain enhancer of activated B cells’ (NF-κB) signaling pathway has been observed throughout the course of CAVS in both VECs and VICs. Inhibition of NF-κB signaling, particularly through the genetic deletion of p65-mediated inflammatory endothelial-to-mesenchymal transition (EndMT), has been shown to attenuate CAVS by reducing VEC dysfunction and VIC calcification [43]. Despite these insights, pharmacological interventions targeting specific pathways involved in the pathogenesis of CAVS have yet to demonstrate significant efficacy in improving aortic valve stenosis [44].

Over the past decade, GLP-1Ras have demonstrated significant cardiovascular benefits in patients with type 2 diabetes, substantially reducing the risk of cardiovascular morbidity and mortality [45]. Their cardiovascular benefits are mainly driven by a significant reduction in the risk for atherosclerotic cardiovascular disease components, mainly stroke, whereas no significant effect on the risk for heart failure (HF) has been demonstrated, with some concerns been raised regarding their use in HF with reduced ejection fraction [46]. To date, there is no evidence concerning the effect of GLP-1Ras on valvular heart disease and related outcomes.

Theoretically, GLP-1Ras could potentially ameliorate several pathways implicated in CAVS pathogenesis. GLP-1Ras are believed to exert atheroprotective effects by attenuating both systemic and localized arterial wall inflammation. These agents have been shown to downregulate the transcriptional expression of genes involved in inflammatory responses and oxidative stress. For example, semaglutide has demonstrated the capacity to lower plasma levels of key pro-inflammatory cytokines, such as interferon-γ (IFN-γ) and tumor necrosis factor-α (TNF-α). Additionally, it reduces mRNA expression of other inflammatory mediators, including interleukin-6 (IL-6), chemokine ligand 2 (CCL2), and vascular cell adhesion molecule-1 (VCAM-1)—all of which are essential for leukocyte recruitment and extravasation [47]. Additionally, GLP-1Ras can inhibit the transcriptional activity of nuclear factor kappa B (NF-κB) and superoxide dismutase 2 (SOD2), crucial regulators of inflammation and oxidative stress. By reversing hyperglycemia-induced DNA demethylation, these agents mitigate oxidative stress and inflammation, thereby offering protective effects against metabolic and vascular complications [48].

Liraglutide has been shown to confer resistance against inflammation induced by TNF-α and lipopolysaccharides while also inhibiting monocyte adhesion to the vascular endothelium by downregulating the expression of VCAM-1 and E-selectin in cultured human aortic endothelial cells [49]. This anti-inflammatory effect is thought to be mediated through the activation of calcium/calmodulin-dependent protein kinase I (CaMKI) and cAMP response element-binding protein (CREB), along with the induction of calcium/calmodulin-dependent protein kinase kinase-β (CaMKKβ) and AMP-activated protein kinase (AMPK) signaling pathways [49]. Additionally, GLP-1Ras have been shown to activate the extracellular signal-regulated kinase 5 (ERK5) pathway, which increases the expression of Krüppel-like factor 2 (KLF2) and mitigates the inhibition of mitogen-activated protein kinase (MAPK). These mechanisms collectively exert anti-inflammatory effects, including reduced leukocyte adhesion [50]. Moreover, GLP-1Ras protect against hyperglycemia-induced autoinflammatory damage by inhibiting the formation of the NLR family pyrin domain-containing 3 (NLRP3) inflammasome, thus providing anti-pyroptotic protection to cardiomyocytes [51]. GLP-1Ras have been shown to significantly reduce systemic inflammatory markers, contributing to their potential cardiovascular protective effects [52]. The effective regulation of inflammation is widely recognized as a key factor in preventing cardiovascular diseases [53]. GLP-1Ras exert anti-inflammatory effects that attenuate the progression of atherosclerotic plaque lesions through multiple mechanisms [54]. Firstly, GLP-1Ras inhibit the expression and release of pro-inflammatory cytokines, such as interleukin-6 (IL-6) and tumor necrosis factor-alpha (TNF-α), thereby dampening the overall inflammatory response [55]. Additionally, GLP-1Ras suppress the activation of nuclear factor kappa B (NF-κB), a central transcription factor involved in the regulation of inflammatory pathways [55]. Inhibiting the NF-κB signaling cascade results in the reduced production of inflammatory mediators, such as adhesion molecules and chemokines, which are critical for the recruitment of immune cells to sites of inflammation. Furthermore, liraglutide has been shown to delay the development of atherosclerosis by inducing cell cycle arrest in vascular smooth muscle cells via the activation of the AMPK pathway [56]. Collectively, these anti-inflammatory properties of GLP-1Ras underscore their potential in mitigating cardiovascular risk by modulating both systemic inflammation and atherosclerotic progression.

Although GLP-1Ras have putative anti-inflammatory effects, imaging studies have yielded conflicting results regarding their effectiveness in reducing vascular inflammation. In a positron emission tomography (PET) study using a preclinical model, semaglutide was found to suppress macrophage activation and metabolic activity within the aortic wall, as evidenced by [64Cu]Cu-DOTATATE and [18F]FDG radiotracers, respectively [57]. However, in the LIRAFLAME trial, liraglutide did not demonstrate a significant reduction in vascular inflammation compared to the placebo, as measured by [18F]FDG PET, following 26 weeks of treatment in individuals with type 2 diabetes [58]. These inconsistencies underscore the necessity for additional human imaging research to more conclusively clarify the impact of GLP-1Ras on atherosclerotic plaque burden and composition.

Regulating lipid levels is essential for mitigating the risk of aortic valve calcification [59]. GLP-1Ras influence multiple aspects of cholesterol homeostasis at both genetic and protein levels. They downregulate lipogenic gene expression while promoting lipolysis in human adipocytes, thereby reducing systemic obesity [60]. GLP-1 receptor activation promotes cholesterol efflux from foam cells through the ATP-binding cassette transporter A1 (ABCA1) [61]. Additionally, GLP-1 Ras reduce the expression of acetyl-CoA acetyltransferase 1 (ACAT1) [62] and enhance signaling between the adaptor protein APPL1 and adiponectin, thereby inhibiting the formation of foam cells [63]. Although the specific effects of GLP-1 Ra monotherapy on lipogenesis have not been fully investigated, co-administration with glucagon has been shown to suppress lipogenesis by downregulating the expression of β-hydroxy β-methylglutaryl-CoA (HMG-CoA) reductase and sterol regulatory element-binding protein-1c (SREBP-1C) [64]. Moreover, GLP-1/glucagon co-agonism enhances reverse cholesterol transport and regulates bile acid homeostasis by increasing the expression of LDL receptor (LDLR), ABCA1, cytochrome P450 7A1 (CYP7A1), and ATP-binding cassette subfamily B member 11 (ABCB11) [64]. Finally, GLP-1 Ras may offer therapeutic potential for nonalcoholic steatohepatitis (NASH) by reducing hepatic inflammation, steatosis, and fibrosis, although the exact mechanisms underlying these effects remain to be elucidated [65].

Despite the multiple beneficial pleiotropic effects of GLP-1Ras against hyperglycemia, adiposity, and inflammation, factors directly implicated into the pathogenesis of CAVS [5,6,7,9], our Mendelian randomization analysis failed to show a significant benefit with GLP-1Ra against CAVS development and progression. A potential explanation for the non-significant effect of GLP-1Ra on CAVS risk could stem from the complex pathophysiology of CAVS, which involves multiple mechanisms beyond inflammation and metabolic regulation, where GLP-1 receptor agonists have their primary effects [23,24,25]. While GLP-1Ras are known for their anti-inflammatory and cardioprotective effects [27], CAVS progression is largely driven by calcification processes that may not be directly influenced by GLP-1 signaling pathways. Additionally, the genetic approach used in this analysis may not fully capture all contributors to CAVS development, particularly those related to environmental exposures. However, it should be highlighted that GLP-1Ras are a class of antidiabetic drugs with the most prominent anti-hyperglycemic effect and based on the crucial role of glycemic control in the progression of CAVS, it appears that GLP-1Ra might indeed be one of the most impactful treatment strategies in patients with concomitant type 2 diabetes and CAVS [66]. Of course, it also remains to be determined whether the significant weight-lowering effects of GLP-1Ras could have a negative impact on CAVS-related outcomes, based on the “obesity-paradox” phenomenon observed in patients with CAVS and type 2 diabetes [67,68]. Therefore, we recognize that GLP-1Ra might theoretically be beneficial for patients with CAVS; however, the significant body weight reduction with GLP-1Ra and the absence of a significant effect on HF and related outcomes raise some concerns on the clinical impact of such a therapeutic intervention in this specific patient population.

The strength of this study lies in the utilization of MR, which offers a robust framework for assessing causality in observational data. By leveraging randomly allocated genetic proxies to study the effects of GLP-1Ra, we minimized the potential for confounding and reverse causation biases commonly encountered in traditional observational studies. However, potential limitations should be acknowledged. Firstly, the present analysis focused on assessing the genetically predicted risk of incident CAVS due to the lack of differentiation in CAVS severities within current GWAS data. Yet, it remains uncertain whether incident and progressive CAVS share common causal factors. Secondly, the limited availability of genetic variants for GLP-1Ras restricted the ability to perform more comprehensive sensitivity analyses to account for horizontal pleiotropy. Thirdly, we utilized summary-level genetic associations with CAVS, which precluded us from performing stratified analyses by factors such as sex or diabetes mellitus status. Fourthly, the genetic data primarily originated from individuals of European ancestry, suggesting potential limitations in generalizing these findings to other ethnic populations. Lastly, while the current study primarily focused on the inflammatory mechanisms underlying CAVS, further investigation into other contributory processes, such as calcification and ectopic ossification, is warranted. Future research should aim to integrate these additional pathways to provide a more comprehensive understanding of CAVS pathogenesis, potentially leading to more effective strategies for prevention and treatment.

## 5. Conclusions

In conclusion, despite the established pleiotropic benefits of GLP-1Ras, including their glucose-lowering, anti-inflammatory, and anti-obesity effects, our analysis did not provide evidence of a significant association between GLP-1Ra and a reduced risk of CAVS. These findings suggest that GLP-1Ras may not play a direct role in preventing the onset or progression of CAVS, at least in genetically predisposed individuals. However, given the important role of glycemic control in the pathogenesis of CAVS, it remains plausible that GLP-1Ras could confer cardiovascular benefits in patients with concomitant type 2 diabetes and CAVS. Our findings highlight the need for more targeted research, including clinical trials assessing the efficacy of GLP-1Ra in diverse patient populations with different severities of CAVS and comorbidities such as type 2 diabetes. Additionally, future studies should aim to explore whether the weight reduction induced by GLP-1Ra could potentially offset any protective effects on valvular calcification in certain populations. Further investigation of GLP-1Ra-repurposing to prevent CAVS in the context of clinical trials and real-world studies is warranted, particularly in high-risk individuals with specific genetic profiles.

## Figures and Tables

**Table 1:** Genetic proxies for GLP-1 receptor agonism and estimates for their association with glycated hemoglobin and type 2 diabetes [32].

						Glycated Hemoglobin			Type 2 Diabetes		
SNP	Chr	Position	EA	OA	EAF	Beta	SE	*p*	Beta	SE	*p*
rs10305420	6	39016636	T	C	0.39	−0.051	0.016	1.30 × 10^−3^	−0.032	0.004	5.11 × 10^−14^
rs75151020	6	39031592	C	A	0.09	0.119	0.026	7.08 × 10^−6^	0.041	0.007	1.37 × 10^−9^
rs2268647	6	39043178	T	C	0.52	0.066	0.015	1.51 × 10^−5^	0.021	0.004	4.95 × 10^−8^

Abbreviations: Chr, chromosome; EA, effect allele; EAF, effect allele frequency; OA, other allele; SE, standard error; SNP, single nucleotide polymorphism.

## Data Availability

The data generated in this research will be shared upon reasonable request to the corresponding author.

## Supplementary Materials

The following supporting information can be downloaded at: https://www.mdpi.com/article/10.3390/jcm13216411/s1, Table S1: STROBE-MR checklist of recommended items to address in reports of Mendelian randomization studies [69].
